# Equitable machine learning counteracts ancestral bias in precision medicine, improving outcomes for all

**DOI:** 10.21203/rs.3.rs-3168446/v1

**Published:** 2023-07-27

**Authors:** Leslie A Smith, James A Cahill, Kiley Graim

**Affiliations:** 1 Department of Computer & Information Science & Engineering, University of Florida, 432 Newell Dr, Gainesville, 32611, FL, USA.; 2 Environmental Engineering Sciences Department, University of Florida, 432 Newell Dr, Gainesville, 32611, FL, USA.

**Keywords:** equitable AI, genomics, cancer, ancestry, artificial intelligence

## Abstract

Gold standard genomic datasets severely under-represent non-European populations, leading to inequities and a limited understanding of human disease [1–8]. Therapeutics and outcomes remain hidden because we lack insights that we could gain from analyzing ancestry-unbiased genomic data. To address this significant gap, we present PhyloFrame, the first-ever machine learning method for equitable genomic precision medicine. PhyloFrame corrects for ancestral bias by integrating big data tissue-specific functional interaction networks, global population variation data, and disease-relevant transcriptomic data. Application of PhyloFrame to breast, thyroid, and uterine cancers shows marked improvements in predictive power across all ancestries, less model overfitting, and a higher likelihood of identifying known cancer-related genes. The ability to provide accurate predictions for underrepresented groups, in particular, is substantially increased. These results demonstrate how AI can mitigate ancestral bias in training data and contribute to equitable representation in medical research.

## 1 Introduction

**Fig. 1:**
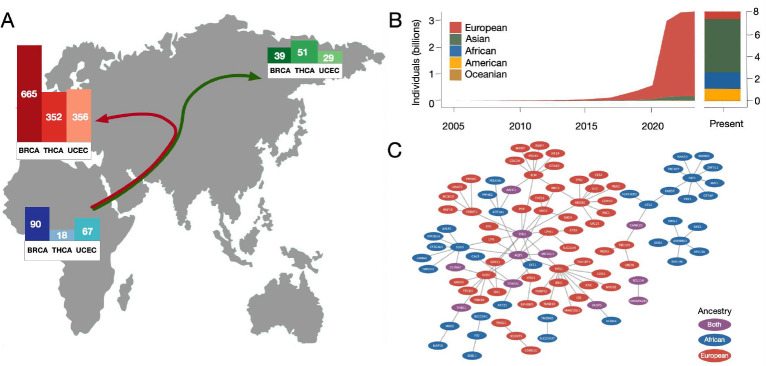
Human diversity and cancer genomics. (**A**) Global map showing the number of samples and sample diversity within three of the most ancestrally diverse TCGA cancers (breast, thyroid, uterine). (**B**) Ancestry statistics for historical GWAS over time, using data from the GWAS Catalog [16]. (**C**) A projection of the EUR BRCA signature of disease (red) versus the AFR BRCA signature of disease (blue) onto a functional interaction network highlights the differences caused by ancestry bias in expression data, signature inter-relatedness, and how this impacts cancer-related precision medicine. EUR and AFR signature overlaps are shown in purple.

Initiatives such as The Cancer Genome Atlas (TCGA) [9–12], All Of Us [13], and Therapeutically Applicable Research to Generate Effective Treatments (TARGET) [14] have generated a wealth of genomic resources for disease analysis, bringing about the field of precision medicine and revolutionizing cancer treatment [15]. However, these databases do not equally represent the diverse human ancestries that comprise the global population [1]. The GWAS Catalog [16], the largest genomic database that explicitly defines ancestry, is currently 95% European ancestry samples, despite Europeans being less than 9% of the global population (Fig. 1B). Numerous recent studies have observed substantial disparities in precision medicine effectiveness across ethnic groups [2–8], demonstrating a fundamental gap and inequity in some of the most cutting-edge approaches to precision medicine for cancer and other genetic disorders.

We address this need with the first ancestry-aware equitable machine learning framework for transcriptomics, PhyloFrame. By incorporating ancestry information, PhyloFrame creates equitable disease signatures that perform well globally, even when trained on non-diverse data. This approach offers an immediately actionable alternative to the mass sequencing that would be required to capture disease-driving genes across underrepresented populations in every cancer. Additionally, PhyloFrame’s inclusion of population structure information inherent to human diversity [17] provides an essential and durable advantage over global bulk analyses regardless of the number of samples involved, allowing us to identify population specific variation more readily. We demonstrate that PhyloFrame provides improved predictive capacity in three diverse TCGA cancers (breast (BRCA), thyroid (THCA), and uterine (UCEC)), even in the (expected) scenario where few ancestries are represented in the training data. PhyloFrame exhibits marked improvements in predictive power for people of undersampled ancestry, illuminating a path towards more equitable and effective precision medicine.

## 2 Global population diversity impacts disease genomics

Despite overwhelming genomic similarity, human populations differ in ways that can substantially impact cancer treatment. African populations have the greatest genetic diversity [18], while non-African populations’ genetic diversity is negatively correlated with distance from Africa due to population bottlenecks during the migration out of Africa [19] (Fig. 1A). In addition, as humans migrated throughout Africa and to other areas of the world (e.g., Asia, Europe, and the Americas), populations underwent selection for traits beneficial in their new environments [20–22]. To discern ancestral differences in disease presentation and susceptibility, we must first develop a better understanding of the genetic variation that exists among ancestries [23].

Ascertainment bias from unrepresentative sampling of ancestries is an acute unsolved challenge in major spheres of human disease genomics, including GWAS [24–26] and transcription models [27–29]. While big data genomic resources have revolutionized the study of cancer and other diseases, the systemic imbalance in cancer genomic data collection across human populations is severe. Given that human ancestry has a substantial impact on gene expression (see Fig. 1C) in healthy and disease tissues [30–33], it is essential that we consider population-specific variation when modeling disease in order to improve cancer treatment for all populations. Access to diverse data is critical for creating robust precision medicine and answering scientific questions in the field [2].

## 3 PhyloFrame unifies ancestry-specific disease signatures

**Fig. 2:**
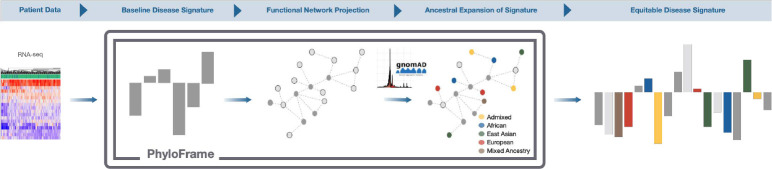
PhyloFrame equitable AI approach. PhyloFrame method takes as input a gene expression matrix of patient disease data and patient outcome labels. Using this information, it calculates an original gene signature, projects that onto the functional interaction network, then identifies closely interacting genes that are enriched in human populations. Using this information, PhyloFrame calculates an updated equitable signature of disease, which is output to the user.

**Fig. 3:**
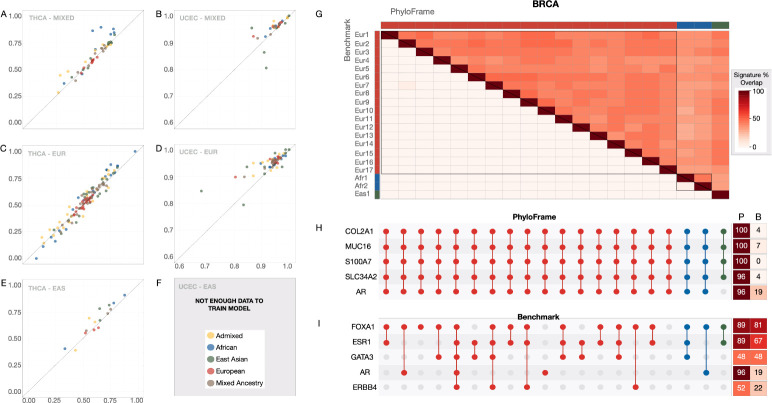
Equitable AI effectiveness. AUC of the benchmark versus PhyloFrame models when training in (**A,C,E**) thyroid cancer (**B,D**) and uterine cancer when using different populations for the training and validation data and varying the ancestral population of the training data. Top row shows results when training using a representative global population, descending rows show training on each cancer using a single human ancestral population. All training sets are randomly sampled from the set of individuals from each ancestry so that all models within each cancer type are trained on the same number of samples. For populations with more samples, we randomly select samples repeatedly until all samples are used in at least one test, resulting in larger numbers of results in different populations. Each scatter plot has AUC of PhyloFrame versus benchmark when testing within an ancestral population. Color coding indicates the test data ancestry and plots are grouped by training data ancestry. (**G**) Signature-signature correlation of BRCA disease signatures when trained on different populations and (**H,I**) Overlap of COSMIC cancer genes in these signatures in the (**H**) PhyloFrame and (**I**) benchmark models. The heatmaps in **H,I** show the number of models in PhyloFrame (P) and benchmark (B) that include the given COSMIC gene.

A recent analysis of cell line data estimated that only 5% of existing transcriptomic data is of African ancestry [34], while another study observed that TCGA cancers have a median of 83% samples of European (EUR) ancestry (range 49–100%) [9]. Recent studies in GWAS data have shown that model efficacy is inversely correlated with population sample size; populations with little or no representation in the data have larger disparities in the disease model performance and garner little benefit from the benchmark disease model [35]. Addressing ancestry imbalance to improve benchmark performance will require several years of dedicated large-scale sequencing efforts to address. Equitable AI approaches can help bridge the gap, and we demonstrate the feasibility of this approach. PhyloFrame, our equitable AI framework, adjusts disease signatures based on population-level genetic variation data (Fig. 2), resulting in ancestry-aware signatures that generalize to all populations, even those not represented in the disease data set. It mitigates the effects of ancestry imbalances in individual disease studies and improves outcomes for all ancestries (Fig. 3A–E).

### 3.1 Ancestry-specific signatures share pathway-level dysregulation

To distinguish ancestry-specific and disease-specific signatures, we first sought to identify differences when training a model using data from different ancestries. We selected the two largest TCGA BRCA populations, European (EUR; 665 samples, 107 basal and 558 luminal) and African (AFR; 90 samples, 37 basal and 53 luminal), and trained an elastic network to predict basal versus luminal breast cancer subtypes. Two models were trained, one using the EUR data and one using the AFR data. Fig. 1C shows the two resultant signatures of disease projected onto the HumanBase mammary epithelium functional interaction network. While there is little direct overlap in the signatures, the network projection highlights the shared pathway level dysregulation and the potential to use functional interaction networks to link together ancestry-specific disease signatures. This suggests that the ancestry-specific disease signatures are strongly interconnected, and that it is possible to create equitable signatures of disease without patient ancestry information. It potentially negates the need for sufficiently large training datasets from each human ancestry population across the globe, necessary to train ancestry-specific models. Instead, it suggests that equitable signatures of disease can be detected from models trained on unbalanced ancestral training data.

### 3.2 Identifying ancestry-specific genetic variants

To guide the network-based signature and eliminate bias toward over-represented ancestries, we identify ancestry specific variants to target during network propagation. We expect that genes with high variance among ancestries are more likely to underly ancestry specific variation, and important disease causing functions will be underrepresented in existing European biased databases. To identify these loci, we define Enhanced Allele Frequency (EAF), a statistic to identify population-specific enriched variants relative to other human populations (see Methods). EAF captures population specific allelic enrichment in healthy tissue, so higher EAF means that individuals from a population are more likely to have a variant than individuals from all other ancestries. Because EAF is calculated from healthy tissue, this approach can integrate information from under-represented populations that are not present in a smaller disease specific databases, including TCGA. To confirm this, we compared EAF in COSMIC cancer-related genes to non-COSMIC genes and did not find an enrichment in EAF (Supplementary Fig. 4; t.test, p-value = 1), as expected, given EAF is calculated from healthy tissue. The average EAF across COSMIC genes is greatest in African ancestry 3.39e−4, whereas in over-represented Europeans average EAF is only −2.87e−5, consistent with the greater genetic diversity found in African compared to other continents [18]. This highlights the importance of broader sampling, as most of the standing variation in humans is derived from under-sampled populations.

### 3.3 Integrating population and disease data

PhyloFrame combines EAF with tissue-specific big-data functional interaction networks to amplify disease-specific information from a user-provided data set (Fig. 2). Analyses in this study uses population-level variation data from gnomAD [36] and tissue-specific functional interaction networks from HumanBase [37]. However, the method accepts alternatives if provided by the user. For any disease, PhyloFrame needs solely the gene expression data and disease subtype information for each sample, allowing for easy application to many diseases. PhyloFrame does not require ancestry information for the training data samples, nor does it assume that individuals are of a single ancestry. Not needing to calculate ancestry is a significant boon; not only is it a computationally intensive task, the methods are recent and constantly evolving. We eliminate one potentially significant bias in equitable AI approaches by not needing to calculate ancestry on the training data and not using it in the model training.

PhyloFrame uses a logistic regression model with LASSO penalty to obtain an initial set of disease-relevant genes. It then projects this set of genes into the HumanBase functional interaction network most applicable to the diseases’ tissue of origin, extending the network to include the first and second neighbors of each gene. These genes are then filtered by EAF, which is used to rank the set of genes identified during network propagation to determine a small set of equitable AI genes. Equitable AI genes identified by EAF are sorted by variation in the training data, and a subset of genes with high EAF and gene expression variability are selected to be added to the equitable AI signature. PhyloFrame then retrains the disease signature regression model and forces the inclusion of the equitable genes using a ridge regression penalty. The resultant signature returned to the user now includes disease-relevant genes that generalize to all populations. We created a benchmark for comparative analyses using the exact LASSO implementation and signature size as PhyloFrame but without network propagation and EAF amplification. See Methods for complete method implementation details of PhyloFrame and the benchmark comparison models.

### 3.4 Ancestry-aware equitable AI disease models improve precision medicine for all

We applied these pan-population generalized signatures to subtype prediction and assessed the predictive power of models trained on different data sets. The breast cancer (BRCA) models were trained to predict Basal versus Luminal subtypes, the thyroid (THCA) models whether a tumor would metastasize (M0 versus MX), and the uterine (UCEC) models Endometrioid versus Serous subtypes. We divided samples from each cancer by ancestry (EUR, AFR, East Asian; EAS, or Admixed; ADMIX). Then we selected a training group size (14–48 samples) such that the smallest ancestry group would still be represented. Multiple models were trained on the over-represented populations to maintain training set sizes while incorporating all individuals. We also employed a ‘Mixed’ ancestry classification wherein each of the ancestry groups above was represented in proportion to their occurrence in the TCGA data. This resulted in many models for some populations, providing a basis for intra-population variation comparisons.

We calculated AUC for each training set under the PhyloFrame and benchmark models to assess predictive power (Fig. 3A–E, Supplementary Fig. 2). In thyroid and uterine cancer, PhyloFrame outperformed the benchmark across training data types. PhyloFrame’s performance advantage is greatest in analyses of the Mixed models (Fig. 3A–B, Supplementary Fig. 2). Despite being trained on a diverse set of samples, the benchmark model has worse performance (mean AUC: THCA: benchmark = 0.63, PhyloFrame = 0.73; UCEC: benchmark = 0.95, PhyloFrame = 0.96) in AFR test data. PhyloFrame’s performance advantage over the benchmark is least pronounced in thyroid cancer models trained on EUR samples (Fig. 3C). However, this effect is stratified relative to overall model performance; the benchmark performs slightly better when both models perform poorly (AUC <0.5), but as model performance improves the AUC differences between PhyloFrame and the benchmark increases, and PhyloFrame becomes more effective (Fig. 3C, mean AUC in >0.5 models PhyloFrame = 0.67, benchmark 0.64). Overall we observe a clear trend toward improved predictive performance in PhyloFrame relative to the benchmark across training sets and test data.

### 3.5 Equitable AI creates more stable disease signatures

Maximizing the amount of model input data is critical to optimizing model stability, minimizing overfitting and identifying a biologically-relevant signature of disease. Doing this requires representative information from all forms of the disease, or if this is not feasible, an understanding that the resultant model will be biased toward what is represented in the data. The stability of disease signatures as the training data is changed is a key metric of model effectiveness. For example, while all of the BRCA models have high AUC (0.99–1), there is significantly less overlap in the disease signatures identified by the benchmark model compared to the PhyloFrame models trained on the same data (Fig. 3G; t-test, p−value=6.494e−06). PhyloFrame models trained on EUR data have, on average, 47% overlap with other PhyloFrame model signatures, whereas benchmark models have 2% overlap with other benchmark model signatures. The greater signature stability between training sets observed in PhyloFrame relative to the benchmark suggests that PhyloFrame is less impacted by overfitting to the training data.

PhyloFrame models are also much more consistent in COSMIC gene identification, identifying 34 unique COSMIC genes (see heatmaps in Fig. 3H,I), compared to 145 COSMIC genes identified by benchmark models. These genes are shared at much higher rates between models in PhyloFrame; 76% COSMIC genes identified in PhyloFrame models are found in multiple signatures compared to only 21% COSMIC genes identified in more than one benchmark signature. Each PhyloFrame BRCA model signature identifies more COSMIC genes than its benchmark counterpart (13 vs 8 COSMIC genes on average; Supplementary Fig. 3, t.test, p−value=1.079e−06). Additionally, the 5 COSMIC genes most frequently identified by benchmark models are more frequently identified by PhyloFrame models (Fig. 3I; 80% vs 50% of models). While the benchmark models most frequently identify canonical BRCA genes such as GATA3 and FOXA1, these are identified by the benchmark at lower rates than they are identified by PhyloFrame (Fig. 3I). COSMIC genes captured by PhyloFrame’s BRCA model (Supplementary Fig. 3) consistently have a higher EAF in ancestries not found in the training data, most significantly in South Asians (t.test, p=1.126e−10). That PhyloFrame also identifies other COSMIC genes at even higher rates than GATA3 and FOXA1 suggests that those genes are deserving of further enquiry for their role in breast cancer, particularly in non-European populations.

## 4 Acute Challenges: Severely underrepresented ancestries

### 4.1 Admixture illuminates precision medicine inequities

**Fig. 4:**
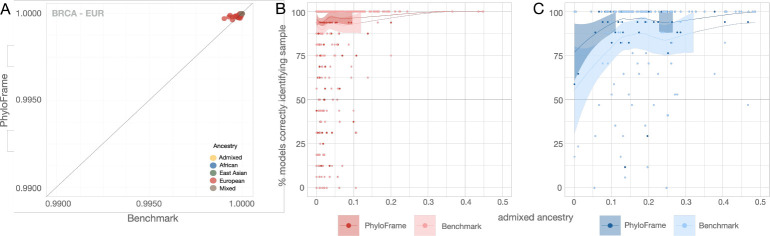
Admixture affects model performance. (**A**) AUC scatter plot of PhyloFrame and benchmark performance in BRCA. (**B-C**) Comparison of models trained on EUR BRCA data and the percent of correctly predicted held-out validation set samples as admixture levels increase in (**B**) PhyloFrame and (**C**) the benchmark model.

Admixed ancestry is widespread and likely to increase in our increasingly interconnected global society. We explored the impact of admixture on model performance in the TCGA BRCA dataset, limiting our analysis to African and European ancestries, (AFR, EUR and ADMIXED individuals with majority African or European Ancestry) as only these groups have a sufficient number of admixed individuals for meaningful analysis. Using models trained on EUR samples, we assessed model recall for PhyloFrame and the benchmark in relation to individual admixed ancestry (Fig. 4, Supplementary Fig. 6). Overall AUC for the benchmark and PhyloFrame is very high (>0.99) for all models and all ancestry test groups (Fig. 4A). Model performance is similarly high and stable across individuals with majority European ancestry, and PhyloFrame slightly outperforms the benchmark across admixture levels (Fig. 4B). However, in individuals with majority African ancestry, PhyloFrame provides significantly higher recall than benchmark models when looking across ancestry levels (Fig. 4C). The performance of both PhyloFrame and the benchmark increase significantly as admixture levels increase. We hypothesize that the improvement in model performance with increased admixed ancestry is a product of ancestral bias in the training data. The preponderance of admixed ancestry in African American populations is European in origin [38], (Supplementary Fig. 1). As such, performance of the models trained on EUR data improves as the individuals’ fraction of European ancestry increases. PhyloFrame does not entirely mitigate this inequity, but it performs substantially better than the benchmark in mitigating the inequalities observed in predicting disease state in individuals with majority African ancestry (Fig. 4C).

### 4.2 External Validation: Triple negative breast cancer in African populations

**Fig. 5:**
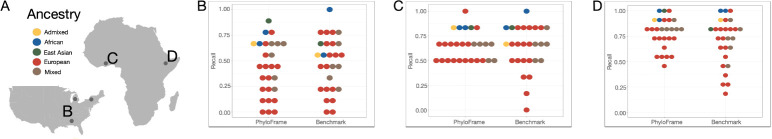
Validation in a global model. ((**A**) A global map of the BRCA validation data sampling sites. PhyloFrame and benchmark model performance in the African breast cancer validation set in each of the sampling sites (((**B**) US, ((**C**) Ghana, ((**D**) Ethiopia).

To externally validate the efficacy of PhyloFrame relative to the benchmark we analyzed triple negative breast cancer (TNBC) data from Martini et al [30], comprised of 9 African Americans, 6 Ghanaians and 11 Ethiopians, totaling 26 patients (Fig. 5A). We chose this dataset for external validation because it provides a unique opportunity to test the efficacy of our models in severely underrepresented populations. Individuals with ancestries that are not well represented in wealthy countries in particular are at greater risk of receiving lower quality precision medicine care under non-ancestry aware methods (e.g. the benchmark). Africa is the most severely impacted continent in this regard due to a combination of economic disadvantages and greater genetic diversity than the rest of the continents combined [18].

Ghanaian and Ethiopian populations are more genetically divergent than any pair of non-African populations [18]. Additionally, the majority of (already underrepresented) African ancestry individuals in genomic datasets are members of the African diaspora [9, 38]. The sizable majority of enslaved people taken to the United States during the transatlantic slave trade were taken from Atlantic Africa, a region including Ghana and several neighboring countries [38–40] (Supplementary Fig. 1). However, it is crucial to stress that Ghanaian and African American ancestry are not equivalent terms; African ancestry in African American populations is diverse and, beyond this diversity, an average of 15% of African American ancestry is European in origin [38]. Under-representation in disease genomics databases is even worse for populations from other regions of Africa, such as Ethiopians, who would be naively classified as African but are genetically very distinct from all of the TCGA training data populations [18]. Ethiopia and nearby countries contribute only 1–2% of the total African American ancestry pool. As such, this ancestry is not evenly distributed across African Americans, the vast majority of whom have little to no East African ancestry, while a small fraction have near 100% East African ancestry [38] (Supplementary Fig. 1).

We applied the trained PhyloFrame and the benchmark models to each validation population to predict TNBC status. Among African Americans, PhyloFrame and benchmark models’ recall was similar when comparing models trained on datasets including individuals with African ancestry (AFR, ADMIX, MIXED). When comparing models trained with no African ancestry individuals, however, PhyloFrame models trained on data that included individuals with African ancestry generally performed better than all other models (Fig. 5B). In African populations PhyloFrame consistently achieves high levels of recall whereas the benchmark produces with both high and very low recall (Fig. 5C–D). This effect is most pronounced in Ethiopians (median recall P 0.82, B 0.73), who are not well represented by any TCGA ancestry groups (Fig. 5D).

## 5 Towards a more equitable precision medicine future

Unbalanced population diversity within accessible genomic data has led to unintended bias in precision medicine models[2, 33, 41–44], resulting in disparate effectiveness in populations and sub-optimal treatment options[5, 7, 45, 46], and contributing to the inequality of medical resources[4, 6, 7, 47–50]. PhyloFrame mitigates these issues through big-data equitable AI, creating genomic signatures of disease that are equally effective in all populations regardless of training data available. This not only brings under-sampled populations to equality with better sampled populations but provides better performance to all populations, including over- and under-represented populations. Integrating population genomic data with disease oncology presents a unique opportunity for novel approaches in the examination of fundamental mechanisms of cancer.

We demonstrate PhyloFrame’s ability to significantly reduce model overfitting caused by ancestral bias in the training data, and to create disease signatures that work better for all individuals. PhyloFrame signatures are more consistent across training sets (demonstrating higher biological relevance) and consistently detect known cancer-related genes. Unlike a comparable benchmark, PhyloFrame is effective regardless of whether an individual comes from a population represented in the training data. It performs well in individuals of divergent unsampled populations and varying levels of admixture.

PhyloFrame occupies a critical, and to this point unfulfilled, niche in the broader movement towards more equitable precision medicine. It serves as a catalyst to improve precision medicine equity, alongside improved genomic references that incorporate population structure and diversity (such as the Human Pangenome Reference Consortium [51, 52]) and sequencing efforts (such as the Nigerian 100K Genome Project [53]). PhyloFrame demonstrates the feasibility of equitable genomic AI approaches. Combined with continually improving genomic resources and data, it will further enable a future of equitable precision medicine, where all individuals can trust that models will accurately predict their genomic information. This represents a major step forward toward a future of equitable genomic AI.

## 6 Materials and Methods

### PhyloFrame equitable AI framework

PhyloFrame provides a flexible platform for ancestry-aware analyses of gene expression data. Here we describe the core features and implementation of PhyloFrame. First, population variation data is downloaded from the selected repository (in this manuscript we use gnomAD v2.1.1, see below). Second, functional interaction network(s) are acquired; we use HumanBase tissue-specific functional interaction networks. These two big-data resources are internal to PhyloFrame. We provide processing scripts to create the correct formats (see Code Availability). PhyloFrame has the option to use user-specified information but these are not required. PhyloFrame defaults to using the HumanBase networks and gnomAD data that we provided in already processed formats. PhyloFrame’s one required input is: gene expression data for a set of patients, and outcome labels with which to train a binary classification model. With this information, PhyloFrame creates a generic disease signature, then uses the population genomics data and functional interaction networks to modify that disease signature to be equally effective across all global populations. PhyloFrame returns this equitable disease signature, including weights describing the model importance for each gene. This process is described in detail below.

### Population Genomics Compendium

PhyloFrame filters genes in each ancestry using sequence variation information from The Genome Aggregation Database (gnomAD) [36]. GnomAD compiles data from many extensive sequencing projects around the globe and is the largest reference population database currently available. The v2.1.1 data set used by PhyloFrame includes 125,748 exome sequences from diverse, unrelated individuals. PhyloFrame uses the the following 17 ancestries: African/African American, Latino/Admixed American, Ashkenazi Jewish, East Asian, European (Finnish), European (non-Finnish), South Asian, and Other. East Asian and European (non-Finnish) are broken down further into East Asian Korean, East Asian Japanese, East Asian Other East Asian, European (non-Finnish) Bulgarian, European (non-Finnish) Estonian, European (non-Finnish) North-Western European, European (non-Finnish) Southern European, European (non-Finnish) Swedish, and European (non-Finnish) Other.

GnomAD information is re-processed using the same pipelines as the gnomAD team, creating a harmonized repository of population-specific genomic information. The gnomAD v2.1.1 exome variant calling format (VCF) file for all chromosomes was downloaded via AWS CLI from gnomAD’s Downloads page. We extract allele frequencies (AF) from gnomAD, for use in our statistical analysis (described below). For all of our analyses we use the full gnomAD dataset, not one of the provided subsets (e.g. pediatric). Variant information for each ancestry as well as the gene information for each Single Nucleotide Polymorphisms (SNPs) were parsed in C++ using the VCF operations library htslib and written to a new VCF file. For each SNP the new VCF contains the allele count, allele number, and allele frequency for each of the 17 ancestries as well as the chromosome, position, rs id, reference allele, alternate allele, gene, consequence, impact, and distance. Allele counts, numbers, and frequencies are not sex specific within the ancestry. The PhyloFrame pipeline for population data is implemented in C++and R.

### Enhanced Allele Frequency

To calculate ancestry enrichment for each SNP, we create a statistic called Enhanced Allele Frequency (EAF). Enhanced allele frequency (EAF) aims to identify Single Nucleotide Polymorphism (SNPs) in exons that vary between global populations. In the current study, EAF is calculated using GnomAD sequence variation data. For each SNP we calculate enhanced allele frequency for each ancestry (17 ancestries total) as follows:

$$\begin{array}{l} EnhancedAF\;Ancestry{\;}_{1}=AF\;Ancestry{\;}_{1}-\;mean\;\left(AF\;Ancestry_{2},\ldots ,\;AF\;Ancestry_{17}\right). \end{array}$$

Enhanced allele frequency shows which SNPs are enhanced, i.e. occur more frequently, in which ancestries and helps PhyloFrame target ancestry-specific genes that may affect the way an ancestry responds to given a disease. High EAF indicates that the given variant is more frequently altered in that ancestry, compared to other ancestries. Density plots of the resulting enhanced frequencies in each ancestry demonstrate that while most SNPs are negatively enhanced, there are ancestry unique peaks of enhanced SNPs (Supplementary Fig. 4).

EAF cutoffs were tested at several thresholds (EX: solely selecting genes with high EAF (0.5 – 1) or selecting genes with low EAF (0.00001 – 0.1)), however PhyloFrame did best with cutoffs between these extremes at 0.001 – 1. The higher threshold prevents highly ancestry specific mutations from being excluded while the lower threshold prevents filtering out major cancer genes, such as FOXA1, that have a relatively lower EAF across ancestries.

### Functional Network Integration

For each disease, HumanBase tissue-specific functional interaction networks [37] were used to find genes likely to interact with the identified disease-associated genes. After the disease relevant tissue-specific network has been downloaded, Entrez IDs are mapped to Gene Symbols in R using Bioconductors’ genome wide annotation for Humans (org.Hs.eg.db, Bioconductor version 3.17).

Genes from PhyloFrame’s baseline elastic net run were used as start nodes in the search for disease relevant gene interactions in the tissue-specific network. First and second neighbors of the baseline signature with a connection between 0.2 – 0.5 are kept for further ancestry allele sorting. By projecting high profile disease associated genes into an interaction network, PhyloFrame is able to begin its ancestry search in genes with high involvement in the disease network.

We trained PhyloFrame on three diseases from the TCGA PanCancer Atlas [9–12]: breast cancer, thyroid cancer, and uterine cancer. These cancers were chosen due to their relatively diverse patient populations. PhyloFrame used the most relevant HumanBase tissue-specific functional interaction networks for each disease: mammary epithelium for breast cancer, thyroid gland for thyroid cancer, and uterine endometrium for uterine cancer. PhyloFrame network cutoffs were initially selected based on a grid search within each disease to find the optimal number of neighbors in the interaction network as well as the optimal edge weight for the interaction. PhyloFrame was run on every combination of the grid N×E where N={1,2,3} defining any gene within three neighbors of a top 20 mutated gene in the disease and E={0.5,0.55,0.6,0.65,0.7,0.75,0.8,0.85,0.9,.95,1} is defining the minimum edge weight needed for a neighbor to be included. PhyloFrame was run on this grid search-for every ancestry in each disease, the combination with the highest overall average across all ancestral models was selected. Based on this search, we opted to included from the functional network the first and second degree neighbors of the original signature genes. We later updated the edge weights to keep nodes with a weight between 0.2–0.5, following analysis of the functional interaction between the European and African TCGA BRCA disease signatures.

### Equitable loss function for machine learning

PhyloFrame builds ancestry unbiased disease signatures by minimizing loss in two domains: relevance to the given disease and enrichment in at least one of the seventeen ancestries described by gnomAD. To define the disease-related genes, we find genes that are functional network interaction partners with the top n most mutated genes related to the disease. Fig. 1C shows the related signatures for breast cancer subtype prediction when training in two ancestral populations. To create the PhyloFrame signatures for this task (results not shown), we created a signature of disease that does not incorporate ancestry, and instead simply calculates relationship within the training data samples to the disease outcomes. We use these genes as start nodes in the search for disease-relevant gene interactions in the tissue-specific HumanBase predicted gene interaction network. These genes are used as a base to put into a tissue-tissue interaction network. PhyloFrame performs a grid search to find fully connected networks within the undirected tissue network graph. Genes are selected if they are within y neighbors of the disease genes, narrowing the genomic landscape by selecting genes enhanced in each ancestry for genes relevant to the disease by looking at the enhanced allele frequency, then logistic regression with a ridge penalty to select critical genes.

### Integrating ancestry and networks

For any disease, PhyloFrame requires the ancestry-specific EAFs for all genes, the tissue associated functional interaction network, patient gene expression data, and patient clinical data. Using the EAF information and functional interaction network, PhyloFrame first finds the neighborhood of the top enhanced genes by EAF, using node and edge parameters previously found in each diseases’ grid search. The resulting neighborhood of genes are mapped to their associated single nucleotide polymorphisms using the previously calculated enhanced allele frequencies. We narrow down the disease’s genomic landscape further by ordering the enhanced allele frequencies for each ancestry separately and targeting the unique allele frequency in each ancestry with the most enhanced SNPs. The genes included in this slice for each ancestry are assembled and prioritized in PhyloFrame’s regression task. To do this, PhyloFrame first narrows the genomic landscape by selecting genes enhanced in each ancestry for genes relevant to disease in each ancestry by looking at the enhanced allele frequency, it then uses logistic regression with a ridge penalty to select highly important genes. It returns to the user an equitable gene signature of disease.

### Data Description

All analyses for this study were conducted on published datasets. Population genomics data from gnomAD (gnomad.broadinstitute.org) was downloaded on July 22, 2022. We downloaded functional interaction networks for model generation from HumanBase (hb.flatironinstitute.org); the mammary network was downloaded on July 5th 2022 and uterine and thyroid networks were downloaded on December 21st 2022. Predictive model training (PhyloFrame and benchmark) and all analyses except the validation experiment (Fig. 5) use gene expression data and matching clinical data from the TCGA Pancancer Atlas for breast (BRCA), thyroid (THCA) and uterine (UCEC) cancers. Data for all TCGA samples was accessed from cBioPortal (www.cbioportal.org) on December 6, 2022 (BRCA), Dec 19, 2022 (THCA), and Dec, 22 2022 (UCEC). Ancestry information for the TCGA cancer data was obtained from Carrot-Zhang et al [33]. TNBC data for the BRCA model validation is from Martini et al [30]. COSMIC data was downloaded on Jan 11, 2023 from the COSMIC website (cancer.sanger.ac.uk/cosmic). It contains 736 genes and information on which cancers each gene has been associated with.

### Data batching for model training

To account for the unequal sample sizes between ancestries, we split the data into batches. To prevent bias when training models due to the disproportionate amount of EUR samples, samples for each disease were split into batches. We also created batches of mixed ancestry, unbalanced ancestry, and single-population ancestry to highlight the effects of PhyloFrame on balanced (unbiased) training data. Number of batches and number of samples in each batch for a given ancestry were determined by the ancestry with the smallest sample size that could still successfully train a model in that disease. All sample sizes must be at least 9 samples and contain at least 4 samples in each subtype. The ancestry with the smallest sample size was used as the base to create other ancestry batches (see Supplementary Table 2 for number of samples per ancestry in each TCGA cancer). The number of samples in an ancestry was divided by the smallest sample size to find the number of batches the given ancestry would be split into. The subtypes in the ancestry were then separated and both were separately divided by the number of batches to find the number of samples from that subtype should be added to each batch. Admixed samples were grouped together regardless of primary ancestry. Overflow samples from each subtype and cancer were added one by one to each batch until all samples were assigned to a batch. For BRCA, a total of 842 basal and luminal samples divided using this approach resulted in 27 total batches; 17 EUR, 2 AFR, 1 EAS, 6 Mixed, and 1 Admixed of 38–48 samples per batch. THCA has data for 436 MX/M0 samples, which resulted in 37 total batches; 23 EUR, 1 AFR, 3 EAS, 9 Mixed, and 1 Admixed batch of 14–18 samples each. UCEC has data for 491 Serous and Endometrial samples total, which resulted in 22 total batches; 12 EUR, 2 AFR, 1 EAS, 6 Mixed, and 1 Admixed batch of 29–39 samples each. No other ancestries had enough samples to train a model, in any of the three cancers.

We used this approach to split the data into equal sized batches of samples, based on the smallest ancestry and cancer type. In total, there are 27 BRCA, 37 THCA, and 22 UCEC training data batches. PhyloFrame and the benchmark models were trained on all sample batches, and applied to the remaining (held out) samples. For BRCA models we also tested on an external validation set [30] (described in External Validation: Triple negative breast cancer in African populations).

### Model Validation Using External Data

To externally validate our model we chose to assess a dataset that both was outside of the training set used in the development of PhyloFrame and that provided an opportunity to assess the performance of PhyloFrame and the benchmark on ancestry groups not present in the training data. To meet these objectives we analyzed triple negative breast cancer (TNBC) data from Martini et al [30], comprised of 9 African Americans, 6 Ghanaians and 11 Ethiopians, totaling 26 TNBC patients (Fig. 5A).

As with the previous analyses of breast cancer, PhyloFrame and the benchmark were tasked with classifying the samples as either basal or luminal. We applied the PhyloFrame and benchmark models trained using the same subdivisions of the TCGA BRCA data described above, resulting in 27 models (17 EUR, 2 AFR, 1 EAS, 1 ADMIXED and 6 MIXED). These trained models were applied to an external validation set, the Martini et al [30] TNBC data. Most basal breast cancers are also TNBCs, and the terms are often used interchangeably. Thus successful models should predict all of the validation set samples to be basal, as they are TNBCs. Because triple negative breast cancers are basal, accuracy functionally reduces to the proportion of the samples in each population that the models correctly identify as basal.

## Supplementary Material

Supplement 1

## Data Availability

This project makes use of data from gnomAD, HumanBase, and TCGA.

## Footnotes

Code availability All code for the PhyloFrame equitable AI model and comparable benchmark model can be accessed at github.com/leslie-smith1112/phyloFrame.
