# Supplementary material for: Equitable machine learning counteracts ancestral bias in precision medicine, improving outcomes for all
Source: Res Sq. 2023 Jul 27:rs.3.rs-3168446. Preprint. [Version 1] doi: 10.21203/rs.3.rs-3168446/v1 (PMC10402189; doi:10.21203/rs.3.rs-3168446/v1)
Supplement: Supplement 1 [file NIHPPrs3168446v1-supplement-1.pdf]

Supplementary information.

Supplementary Information

Model performance in three cancers

Model performance was compared using AUC, recall, and signature overlap. Each metric and associated results are described in the paragraphs below. We tested the efficacy of PhyloFrame against the benchmark for multiple cancer prediction tasks.

Subtype prediction in breast cancer (BRCA)

Cancer subtype prediction is a key component of directing the treatment of breast cancer. We trained 27 models on data from 842 individuals, divided into training batches of 38-48 samples each, to predict Basal vs Luminal subtype. The number of training sets correspond to the proportion of samples in the TCGA BRCA database resulting in 17 EUR, 2 AFR, 1 EAS, 1 ADMIXED and 6 MIXED models. Both PhyloFrame and the benchmark use the HumanBase mammary epithelium network for network construction. PhyloFrame uses population data from gnomAD.

For each model (PhyloFrame or benchmark) we predicted subtypes for entire populations and calculated AUC. If a model was trained on the same population being tested (eg. EUR trained model being tested in EUR), we excluded the samples used for training from the larger set. We plotted AUC results for the benchmark compared to PhyloFrame (Supplementary Fig. 2A-E).

Metastasis prediction in thyroid cancer (THCA)

Metastases have a substantial impact on patient prognosis, in this analysis we predicted whether patients would undergo metastasis (M0 versus MX). We trained 37 models on data from 436 individuals, divided into training batches of 14-18 samples each. The number of training sets correspond to the proportion of samples in the TCGA THCA database resulting in: 23 EUR, 1 AFR, 3 EAS, 1 ADMIXED and 9 MIXED models. Both PhyloFrame and the benchmark use the HumanBase thyroid gland network for network construction. PhyloFrame uses population data from gnomAD.

For each model (PhyloFrame or benchmark) we predicted subtypes for entire populations and calculated AUC. If a model was trained on the same population being tested (eg. EUR trained model being tested in EUR), we excluded the samples used for training from the larger set. We plotted AUC results for the benchmark compared to PhyloFrame (Supplementary Fig. 2F-J).

Subtype prediction in uterine cancer (UCEC)

Cancer subtype prediction is a key component of directing the treatment of uterine cancer. We trained 22 models on data from 491 individuals, divided into training batches of 14-18 samples each, to predict Endometrioid vs Serous subtype. The number of training sets correspond to the proportion of samples in the TCGA UCEC database

resulting in 12 EUR, 2 AFR, 1 EAS, 1 ADMIXED and 6 MIXED models. Both PhyloFrame and the benchmark use the HumanBase uterine endometrium network for network construction. PhyloFrame uses population data from gnomAD.

For each model (PhyloFrame or benchmark) we predicted subtypes for entire populations and calculated AUC. If a model was trained on the same population being tested (eg. EUR trained model being tested in EUR), we excluded the samples used for training from the larger set. We plotted AUC results for the benchmark compared to PhyloFrame (Supplementary Fig. 2K-O).

Model stability and consistency

Precision medicine models should be identifying biologically-relevant signatures of disease. A signature may be accurate on a set of training data, but it has limited utility if it does not generalize to other data or identify the biological drivers of disease. To quantify the amount of biological overlap between signatures, we calculate several factors. First, we identify how many known cancer-related genes are identified in each signature, and which cancers they have previously been associated with. This is done using COSMIC cancer genes (see Fig. 3H,I). We calculate both the number of COSMIC genes identified by each model, and calculate a t-test comparing these numbers in PhyloFrame versus benchmark models. Second, we calculate the overlap in disease signatures for each model. Higher signature overlap is an indication that, despite different training data, the models are identifying the same factors as driving the disease. To quantitatively compare this overlap, we calculate pairwise model signature correlations. Signature correlations are calculated based on presence/absence of each gene in a model signature; we do not consider model weights for the genes. This resulted in a matrix of signatures by signatures, filled in with pairwise signature-signature correlations. PhyloFrame signatures have significantly higher overlap than benchmark models (mean 47% vs 2% overlap, Fig. 3G). We then ran a t-test comparing all PhyloFrame-PhyloFrame model pairwise correlations against all benchmark-benchmark model pairwise correlations, and found there is statistically higher likelihood of signature overlap in PhyloFrame compared to benchmark models.

Sample-specific model performance

Some samples are far more difficult to predict than others, and while overall model AUC is important, it is also valuable to see how each model performs in the more difficult sample sets. To see this, we plot the per-sample differences in model performance (% of models that correctly predict each sample; Supplementary Fig. 5), and identify which samples are often misclassified, if any. Each point in these plots represents one sample. In the boxplots, samples are grouped by ancestry and by model type (PhyloFrame vs benchmark). The y-axis shows the percent of models that correctly predict each sample. Both PhyloFrame and the benchmark models struggle to correctly predict a small subset of samples. For most of the BRCA samples, all models correctly predict BRCA subtype. A small subset of samples are incorrectly predicted by all or most of the BRCA models (see Fig. 4 for a critical factor explaining of this effect. UCEC and THCA models have far more variability, as expected, given

the models have lower average AUC than the BRCA models. Metastasis is a harder prediction task than tumor subtypes, and so many of the THCA models have low performance. Of note, this per-sample performance is not shared across models; There is a wide range of success for each set of sample predictions. This suggests that there are factors relevant to the THCA models that are not being identified by the models. It is unclear if this is a tractable prediction task, given the small training data size. UCEC models similarly have varied performance for each sample, however most samples have a high % prediction success across models. The endometrial versus serous subtype UCEC model predictions for a subset of samples are unreliable. This prediction task is difficult due to the low number of serous samples available in the training data. Serous samples are only approximately 25% of the samples, and half of those samples are from individuals of European decent. For example, an East Asian model could not be trained in uterine cancer because there were only 3 serous samples. The samples most often misclassified in this prediction task are of the serous subtype.

COSMIC gene enrichment and presence

COSMIC currently includes 736 genes expert curated and validated as cancer-related based on previous studies. We used COSMIC in two sets of analyses. First, we used COSMIC genes to identify the EAF trends of disease-related genes, to assess the effectiveness of using EAF as an equity adjustment in AI methods. We found that there is no EAF enrichment in COSMIC versus non-COSMIC genes (Supplementary Fig. 4; t.test, p-value = 1), suggesting that the utility of EAFs in equitable AI is not limited to cancer studies. Second, we used COSMIC genes to determine the extent to which model signatures in this paper recapitulate known cancer processes and as a validation set of genes that ideally will be enriched in the disease signatures. As most of the models in this paper are trained on smaller sample sizes (due to ancestry bias in the data), we expect model overfitting. Identifying the number and variety of COSMIC cancer genes in each signature helps to determine how much of the signatures are cancer-related. COSMIC genes with high EAFs are more frequently enriched in African and East Asian but not European ancestries (Supplementary Fig. 4B; t-test, p-value ¡ 2.2e-16) . For example, FOXA1 is one of the five most frequently identified COSMIC genes by the benchmark BRCA models (Fig. 3H,I). While it is not one of the most frequently identified COSMIC genes by PhyloFrame models, more PhyloFrame (89%) than benchmark models (81%) include FOXA1 in their signatures (22 vs 24 of a total 27 models).

The impact of admixture

Continental and ethnic classifiers, including those used to group samples in this study, are flawed proxies for ancestral diversity. In an increasingly interconnected world, rates of admixed ancestry are likely to increase. Even in the present day, the extent and impact of admixture within human populations remains under-recognized, especially as it pertains to underrepresented groups in the United States. We sought to understand how admixture impacts the predictive power of PhyloFrame relative to the benchmark.

To explore the impact of admixture we examined predictive efficacy of models trained on EUR breast cancer data. We selected breast cancer because the overall high AUC across all models allows us to largely exclude noise generated by poor model performance, and instead identify which individuals the models' struggle to accurately predict. We used EUR training data because having more trained models (in this case, 54 models) enables us to precisely determine whether an individual is being stochastically or systematically incorrectly predicted. The current dramatic over-representation of Europeans in genomic databases [9, 34] provides additional value for this approach as it more closely mirrors expected real world studies across disease types.

We calculated the fraction of models that correctly predicted an individual's subtype and plotted those relative to their admixed ancestry proportion. Note that this includes individuals that are otherwise classified as Admixed (greater than 20% non-majority ancestry) being grouped according to their majority ancestry. Next, we measured the statistical variance of model prediction accuracy across populations (R geom_smoothing with LOESS model). Individuals with majority European ancestry show stable prediction across admixture levels (Fig. 4B), compared to PhyloFrame improvements on individuals from many ancestries and models, most notably the BRCA EUR models applied to Admixed and AFR samples (Supplementary Fig. 5). Majority African ancestry individuals show both significant increases in model performance with increasing admixed ancestry and significantly better performance in PhyloFrame than in the benchmark. Given that the vast majority of admixed ancestry in African Americans is European, including in the individuals in this study (Supplementary Table 2), this highlights a shortcoming of current predictive methods that is easily overlooked when grouping individuals by continental level ancestry or by overall model AUC and other performance metrics. Overwhelmingly European training data sets may appear to perform acceptably in African ancestry individuals, when in fact performance is not uniformly high across the group. This raises substantial concerns surrounding existing models' abilities to provide insightful and accurate precision medicine predictions for individuals of un-admixed African ancestry.

External validation of BRCA models

To externally validate our model we chose to assess a dataset that both was outside of the training set used in the development of PhyloFrame and that provided an opportunity to assess the performance of PhyloFrame and the benchmark on ancestry groups not present in the training data. To meet these objectives we analyzed triple negative breast cancer (TNBC) data from Martini et al [30], comprised of 9 African Americans, 6 Ghanaians and 11 Ethiopians, totaling 26 TNBC patients (Fig. 5A).

Data and preprocessing

As with the previous analyses of breast cancer, PhyloFrame and the benchmark were tasked with classifying the samples as either basal or luminal. We applied the PhyloFrame and benchmark models trained using the same subdivisions of the TCGA BRCA data described above, resulting in 27 models (17 EUR, 2 AFR, 1 EAS, 1

ADMIXED and 6 MIXED). These trained models were applied to an external validation set, the Martini et al [30] TNBC data. Most basal breast cancers are also TNBCs, and the terms are often used interchangeably. Thus successful models should predict all of the validation set samples to be basal, as they are TNBCs. Because triple negative breast cancers are basal, accuracy functionally reduces to the proportion of the samples in each population that the models correctly identify as basal.

Model results

In African populations (Ghanaian and Ethiopian), all but one PhyloFrame model have performance greater than random chance accuracy (>50%). Mean PhyloFrame performance is higher than the benchmark model in both the Ghanaian validation set samples (mean recall PhyloFrame = 0.64 vs benchmark = 0.62) and the Ethiopian validation set samples (mean recall PhyloFrame 0.78 vs benchmark 0.70). Performance is more similar in the African American validation set samples (mean recall PhyloFrame = 0.42 vs benchmark 0.47).

Martini et al samples from the US were collected from New York City (New York), Detroit (Michigan), Ann Arbor (Michigan), and Birmingham (Alabama). TCGA samples also were recruited from the US, and TCGA BRCA samples came from 42 tissue source sites, including several in New York City. Given that the training data includes samples from overlapping populations, it was expected that the benchmark model would perform well in the African American validation set samples, however this is not the case (median = 0.56). Both the benchmark and PhyloFrame models have highly variable performance in the validation set African American samples, suggesting that neither set of models are able to fully disentangle complexities of ancestry and breast cancer subtypes. Given that the Basal subtype is enriched in African Americans [30, 42, 54], this prediction task may be intrinsically connected to ancestry; It has been previously suggested that the Basal/Luminal subtypes are unintentionally linked to African ancestry. However, there are distinctions between the two datasets, even within African American samples, that may explain some of the variability. TCGA BRCA samples were diagnosed with BRCA from 1988-2003, compared to the BRCA validation set which began collection in 2006. Ghanaian samples average age 48 years, Ethiopian 41 years, and the African American samples 68 years [30], compared to 58 years for the TCGA BRCA samples. The age differences may account for some of the variance in the PhyloFrame and benchmark models' recall when applied to the African American samples.

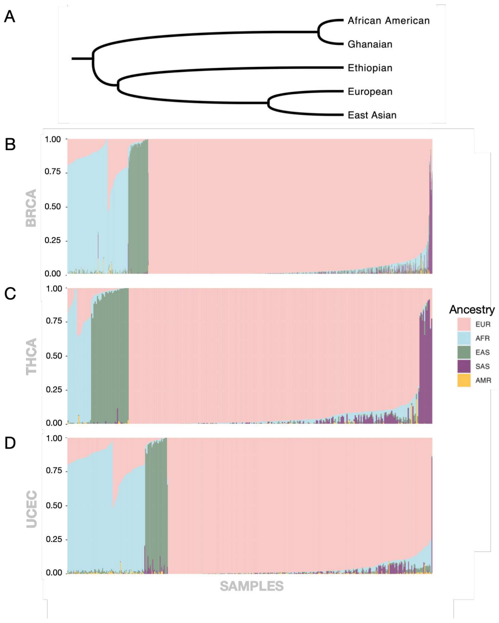

Supplementary Figure 1 African ancestry diversity. (**A**) Phylogenetic tree showing the general pattern of genetic relationships between the populations used for model training and disease state prediction in this study. (**B-D**) Estimated ancestry of each patient in the TCGA data for (**B**) BRCA, (**C**) THCA, and (**D**) UCEC. Genetic ancestry for each TCGA sample was computationally predicted by Carrot-Zhang et al [33], who used 5 ancestry-calling pipelines to generate ensemble predictions of each individual in the TCGA PanCancerAtlas. Ancestry sample counts across all TCGA cancers shown in Supplementary Table 2. Each column represents a single sample in the cohort, and the colored bar represents the estimated amounts of ancestry in each of the major global populations. Shown populations are EAS (green), SAS (purple), AFR (blue), EUR (pink), and AMR (yellow).

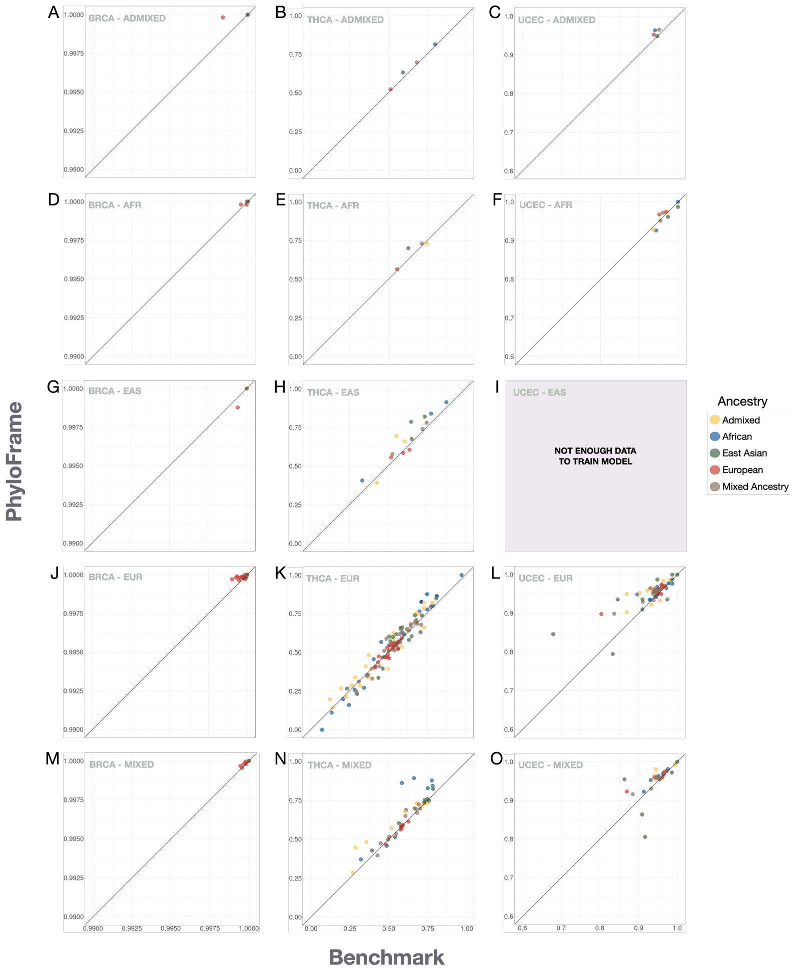

Supplementary Figure 2 Equitable AI effectiveness. AUC of the benchmark versus PhyloFrame models when training in (**A-E**) BRCA, (**F-J**) THCA, and (**K-O**) UCEC using different populations for the training and validation data and varying the ancestral population of the training data. Rows correspond to training data used (ADMIXED, AFR, EAS, EUR, MIXED). MIXED indicates that the training data ancestry diversity matches that of the TCGA data; it is not representative of the global population distributions. Each dot coesponds to a combination of training and test data, color coded by test data.

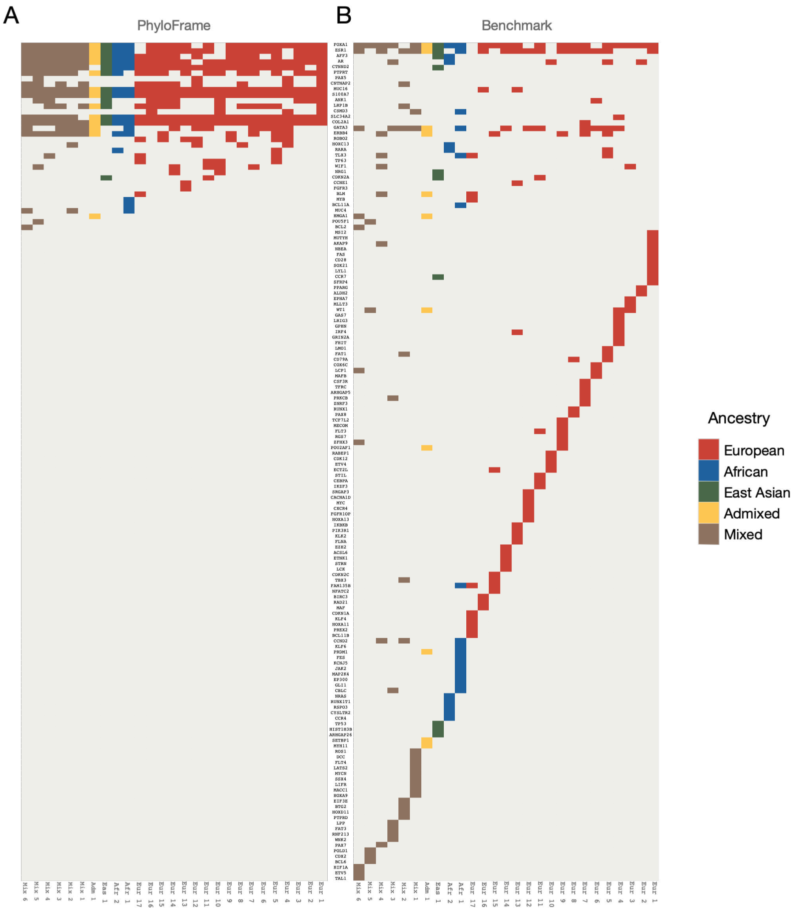

Supplementary Figure 3 COSMIC gene enrichment in model signatures An indicator plot showing (**A**) PhyloFrame and (**B**) benchmark model signatures and which COSMIC genes are included in each. Each column represents one trained model and each row is a COSMIC gene. Indicator marks are colored by the ancestry of the training data for the model.

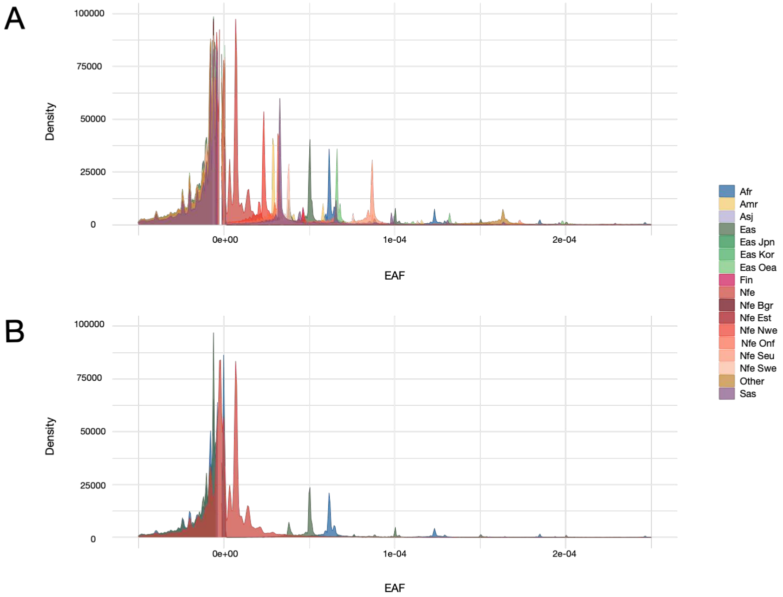

Supplementary Figure 4 Transcriptome-wide EAF enrichment EAF density plots for (**A**) all genes and (**B**) COSMIC cancer genes, grouped by ancestry. **A** shows all 17 gnomAD ancestries and **B** shows the EUR, EAS, and AFR ancestries used to group the ancestry-specific training data sets for the AI models. Peaks demonstrate unique EAF across ancestries.

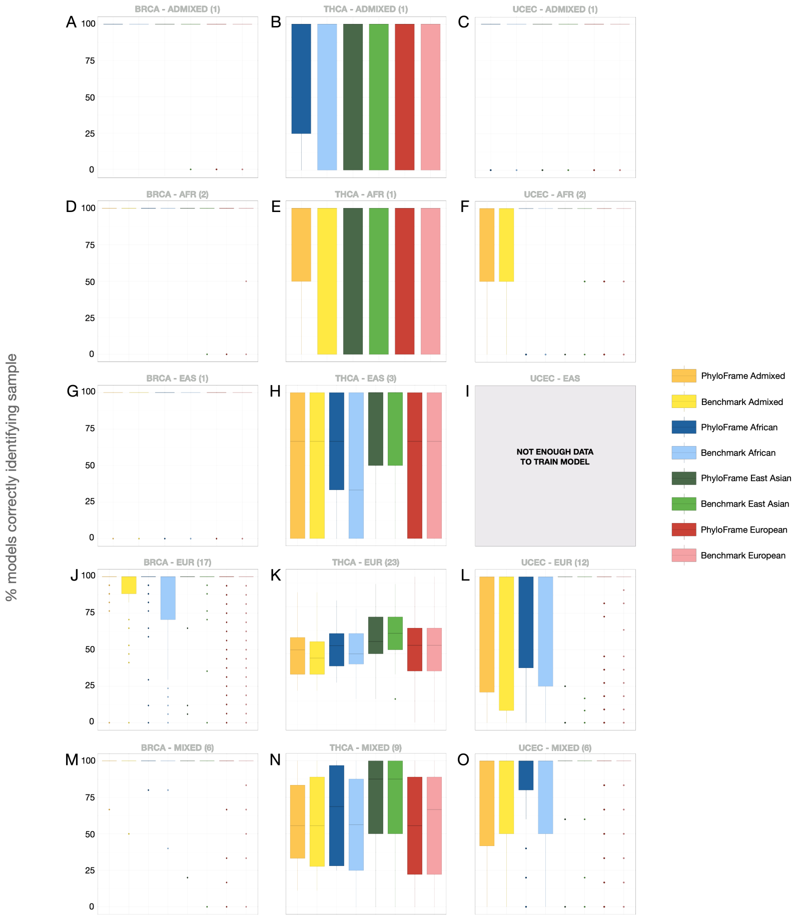

Supplementary Figure 5 Sample-specific model performance To ascertain which, if any, samples are harder to predict, we calculated performance of all models for each sample. Boxplots show the percent of models that correctly subtype each sample in (**A,D,G,J,M**) BRCA, (**B,E,H,K,N**) THCA, (**C,F,I,L,O**) UCEC. Samples are grouped by genetic ancestry and model type (PhyloFrame or benchmark). The EAS UCEC plot is greyed out as there are not enough samples to train the models. In each plot, each dot is a single sample and y-axis shows percent of models that correctly classify that sample.

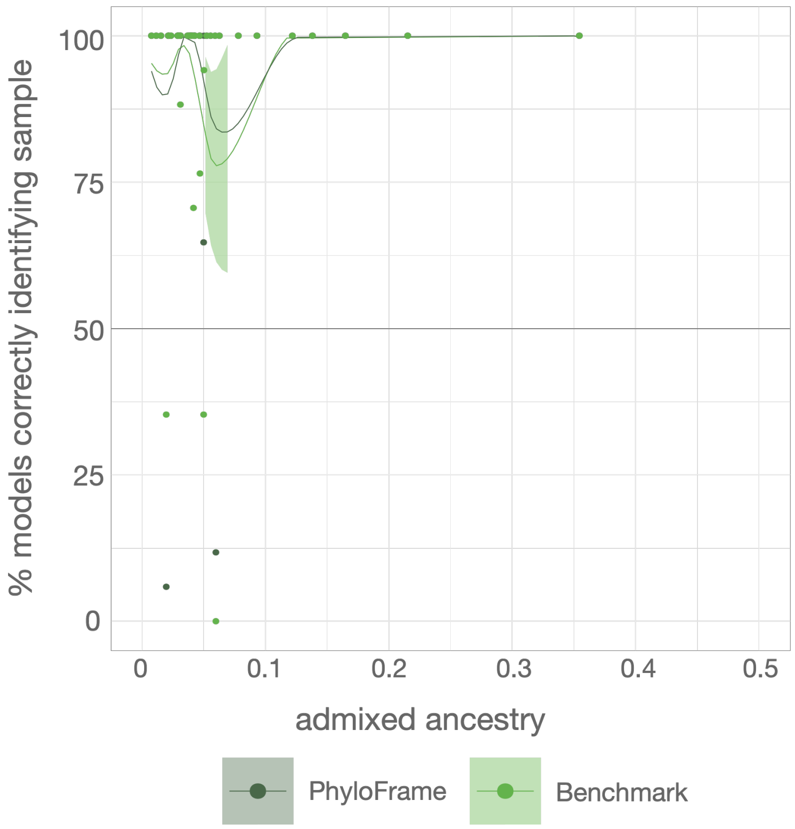

Supplementary Figure 6 Effect of admixture on EUR-trained model performance. A comparison of models trained on EUR BRCA data and the percent of correctly predicted held-out EAS BRCA samples as admixture levels increase in PhyloFrame (dark green) and benchmark (light green) models.

TumorType	admix	AFR	AFR_Admix	AMR	EAS	EAS_Admix	EUR	EUR_Admix	SAS	SAS_A
ACC	0	0	2	0	2	0	81	1	0	0
BLCA	0	14	7	1	43	0	328	1	1	1
**BRCA**	1	125	56	5	56	1	822	3	4	4
CESC	0	19	12	1	21	1	194	15	0	0
CHOL	0	2	0	0	2	0	29	1	0	0
COAD	0	46	14	0	12	0	380	2	0	0
DLBC	0	1	0	0	15	1	30	0	1	0
ESCA	0	3	1	0	44	0	123	4	0	0
GBM	1	24	21	0	6	0	450	5	0	3
HNSC	1	38	12	6	6	1	437	4	3	2
KICH	0	3	1	0	1	0	56	0	0	1
KIRC	0	32	23	2	7	1	432	6	0	1
KIRP	0	43	20	0	6	0	208	1	1	0
LAML	0	16	0	0	2	0	178	0	0	0
LGG	0	15	8	4	10	0	449	7	1	2
LIHC	1	14	4	0	163	0	177	3	0	1
LUAD	0	42	18	1	9	0	502	0	0	0
LUSC	0	15	15	0	11	0	455	3	0	0
MESO	0	0	0	0	0	0	82	0	0	1
OV	0	23	11	1	15	1	504	1	2	4
PAAD	1	6	3	0	11	0	158	0	0	0
PCPG	0	8	12	0	3	0	146	0	4	0
PRAD	1	38	21	0	9	0	409	2	0	2
READ	0	1	5	0	1	0	155	1	0	0
SARC	2	12	6	0	6	0	220	1	0	0
SKCM	0	1	0	2	12	0	448	0	0	0
STAD	0	8	7	0	90	0	294	0	0	0
TGCT	0	1	3	0	4	0	121	1	0	0
**THCA**	3	21	12	11	53	0	364	1	9	2
THYM	0	4	4	0	12	0	92	4	0	0
**UCEC**	1	72	40	7	34	1	389	1	1	0
UCS	0	4	5	0	3	0	43	0	0	0
UVM	0	0	0	0	0	0	80	0	0	0

Supplementary Table 2 TCGA Cancer samples per ancestry. Summary of samples per ancestry across all TCGA can BRCA has by far the most samples and is the most diverse cancer in the TCGA dataset. AMR, SAS, and admix individuals ar severely underrepresented with only 41 AMR, 27 SAS, and 12 admix individuals across all cancers.
